# Severe reinfection with severe acute respiratory syndrome coronavirus 2 in a nursing home resident: a case report

**DOI:** 10.1186/s13256-021-02958-4

**Published:** 2021-07-20

**Authors:** Nimrah Bader, Mahmood Khattab, Fahmi Farah

**Affiliations:** 1 Department of Internal Medicine, University of Oklahoma Health Sciences Center, 800 Stanton L Young Blvd, Suite 6300, Oklahoma City, OK 73104 USA; 2 Baylor Scott & White, Heart and Vascular Hospital, 7100 Oakmont Blvd, Suite 201, Fort Worth, Dallas, TX 76132 USA

**Keywords:** COVID-19, Immunity, Reinfection, SARS-CoV-2

## Abstract

**Background:**

The topic of natural immunity related to severe acute respiratory syndrome coronavirus 2 remains controversial. Although evidence suggests postinfection immunity can be achieved, there have been reported cases of reinfection with similar or milder symptoms. Information on severe disease manifestation during reinfection is not known. We present a case of reinfection with a more severe presentation as compared with the initial infection.

**Case Report:**

We describe a white male patient from a nursing home who was reinfected with severe acute respiratory syndrome coronavirus 2 with severe disease manifesting as dyspnea, fevers, and encephalopathy with hypoxemic respiratory failure requiring intubation, elevated inflammatory markers, and lung infiltrates on imaging, after initially testing positive with mild symptoms 2 months prior to presentation. Notably, severe acute respiratory syndrome coronavirus 2 antibodies were detected, which indicated this was a coronavirus disease 2019 reinfection. After treatment with remdesivir, dexamethasone, and convalescent plasma, he was subsequently extubated and discharged home after 2 weeks.

**Conclusion:**

It is not clear whether an initial infection with severe acute respiratory syndrome coronavirus 2 and recovery provides prolonged immunity beyond 2 months. Furthermore, even if antibodies are present, it does not guarantee an attenuated course during reinfection. Therefore, vaccination plays an important role in prevention. Long-term cohort studies will be needed to study the factors behind reinfection.

## Background

In January 2020, the World Health Organization described a novel coronavirus severe acute respiratory syndrome coronavirus 2 (SARS-CoV-2) from Wuhan, China, causing viral pneumonia later named as coronavirus disease 2019 (COVID-19), which shortly became a global pandemic [1]. At the time of writing, more than 76,000,000 global cases have been reported with 1,691,000 deaths [2]. The topic of immunity has garnered widespread interest in vaccines. Presently, three vaccines are available and recommended for use in the USA [3].

The topic of natural active immunity as it pertains to SARS-CoV-2 remains controversial. It is unclear whether infection with SARS-CoV-2 can provide long-lasting immunity. Although evidence suggests postinfection immunity can be achieved [4], there have been reported cases of reinfection [5–7]. This raises the important question of whether long-lasting immunity can be achieved and, if so, whether it protects against a severe form of the disease or provides complete immunity.

In this case report, we describe a patient who was admitted with severe hypoxemic respiratory failure secondary to COVID-19 after initially testing positive with mild symptoms 2 months prior to presentation.

## Case

**Fig. 1:**
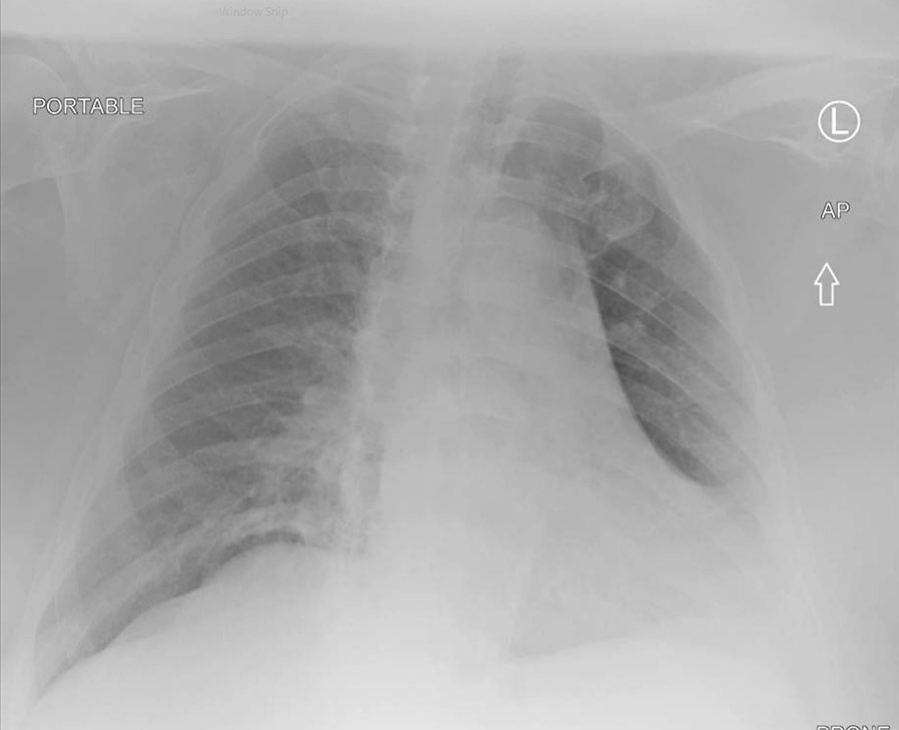
Initial Chest X-Ray in April 2020 with coronavirus disease 2019 infection

In April 2020, a 73-year-old white male nursing home resident with known history of obesity, chronic obstructive pulmonary disease (COPD) on chronic oxygen supplementation on 2 L with rest, obstructive sleep apnea, stroke, pancreatic insufficiency, and type II diabetes mellitus presented with shortness of breath and a positive COVID-19 nasal swab polymerase chain reaction (PCR) test. Vitals were within normal limits, and he was saturating 96% on 2 L nasal cannula, which was his baseline. Pertinent laboratory results included white blood cell (WBC) 6.4 K/mm^3^, Absolute Neutophill count (N#) 4.1 K/mm^3^, Absolute Lymphocyte count (L#) 1.5 K/mm^3^, lactate dehydrogenase (LDH) 131 unit/L, C-reactive protein (CRP) 5.5 mg/L, and ferritin 221 ng/ml. Kidney and liver function tests remained within normal limit. Chest x-rays showed an obese body habitus with mild-to-moderate cardiomegaly and clear lung fields (Fig. 1). This was similar compared with his previous chest x-rays since 2018. He was monitored in the hospital and discharged after 3 days. Two nasal PCR tests were negative; one performed on the day of his hospital discharge and the second one repeated after 14 days of discharge.

**Fig. 2:**
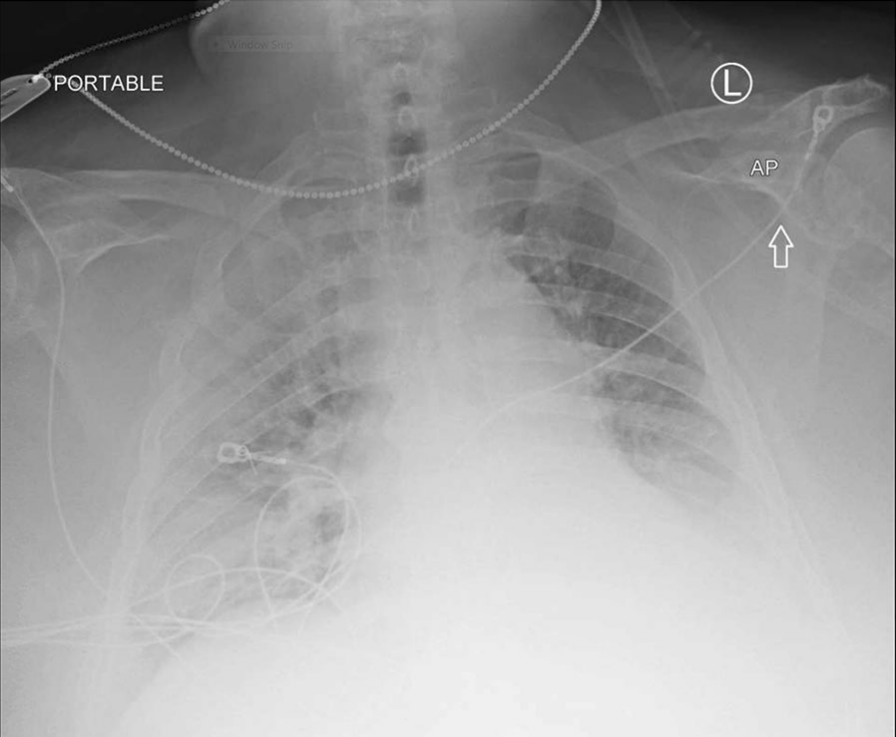
Chest X-Ray in June 2020 with coronavirus disease 2019 reinfection

**Fig. 3:**
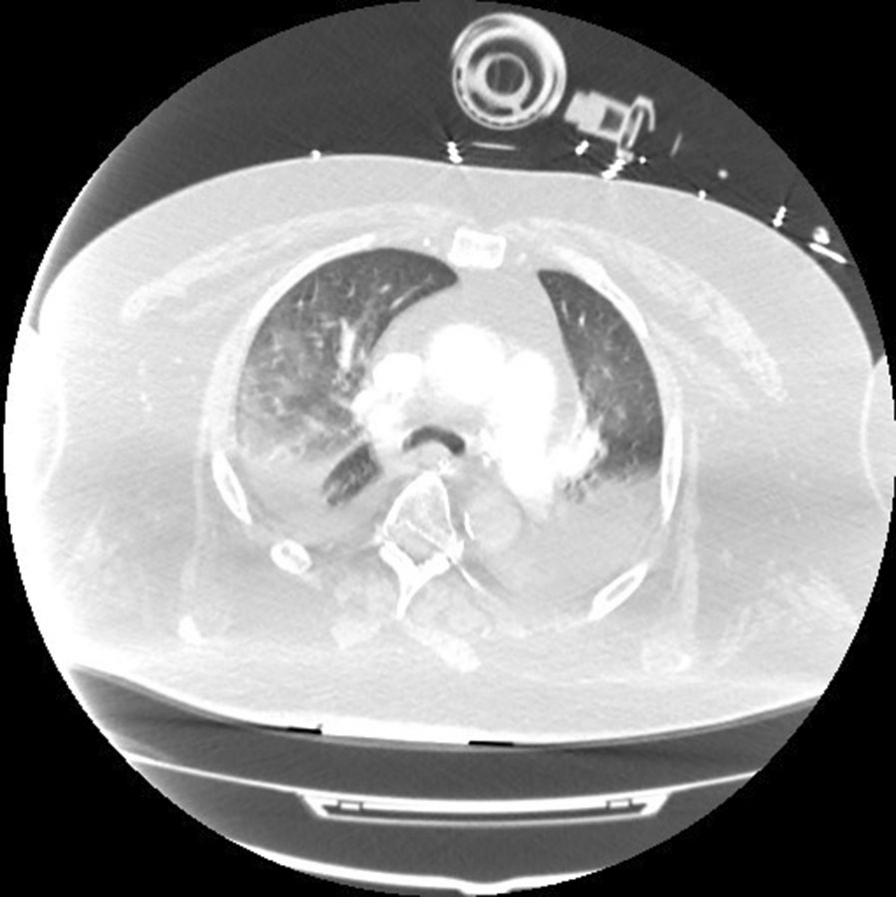
Computed tomography chest transverse view in June 2020

**Fig. 4:**
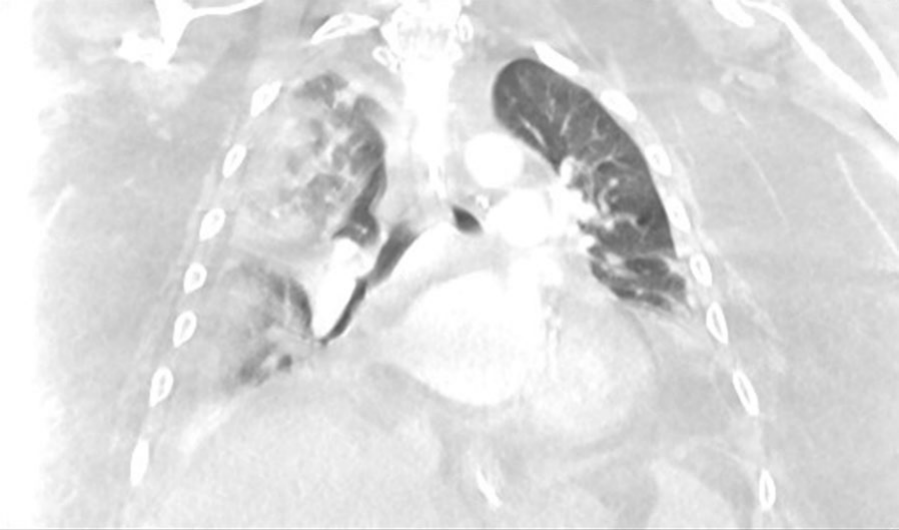
Computed tomography chest coronal view in June 2020

Two months later, in June 2020, he presented to the hospital again, this time with progressive dyspnea, subjective fevers, and confusion. Vital signs were remarkable for blood pressure (BP) 96/52 and SpO_2_ 60% on arrival. Examination was notable for encephalopathy; auscultation was limited by body habitus. He was initially placed on bilevel positive airway pressure (BiPAP) with improvement in his oxygen saturation. Initial arterial blood gas (ABG) was 7.22/107/52 on 0.4 FiO_2_, confirming he was in acute hypoxemic and hypercapnic respiratory failure. His COVID-19 PCR test was positive on admission. Of note, SARS-CoV-2 IgG antibodies were detected on assays on admission. Chest x-ray was notable for diffuse infiltrates (Fig. 2). Computed tomography (CT) scan of the chest showed bilateral pleural effusions with consolidation or atelectasis in the lower lobes and patchy air space opacities involving the right upper lobe (Figs. 3 and 4). He was intubated because of persistent hypoxemia. He was treated with a 10-day course of remdesivir, convalescent plasma, and dexamethasone 6 mg daily. Piperacillin–tazobactam was also started to cover for bacterial pneumonia. He was extubated after 4 days and discharged back to the nursing home after 2 weeks in the hospital. Before discharge, two COVID-19 PCR tests were done, which were negative. At the time of writing this case report, he remains at his nursing home and has received his COVID-19 vaccine.

## Discussion

Our case highlights that reinfection with COVID-19 is possible, like in previous case reports; where it differs is that it described a more severe reinfection, while others described the reinfection to be similar or less severe than the initial presentation [5–8].

For instance, in a study where 220 patients that initially tested positive for COVID-19 were followed closely for 2 months after discharge, 27 tested positive on throat swab viral RNA at follow-up [9]. Of this group, there were two patients that were considered to have true reinfection as they had recovered clinically from the virus, had a negative throat swab viral RNA test afterwards, and were subsequently hospitalized with positive throat swabs. During their second hospitalization with the infection, the severity of their illnesses was classified as similar to their initial hospitalizations. In contrast, the patient we present initially tested positive on viral RNA PCR, was hospitalized with a mild severity of infection, and discharged back to his nursing home with a negative nasopharyngeal swab after recovering clinically. He returned 2 months later with worsening symptoms, a positive PCR test, and positive antibodies, which likely indicated reinfection. Furthermore, he returned with a more severe infection, requiring intubation and mechanical ventilation.

Immunity following infection as it pertains to the SARS-CoV-2 virus has been studied, with the knowledge that specific antibodies and cell-mediated responses are induced. The question that continues to be studied is whether these responses provide long-term protective immunity and whether they provide sterilizing immunity or simply protect from a severe disease course [10]. Studies have identified that there are both CD4 and CD8 T-cell responses in patients who had recovered from COVID-19 and in individuals who had received an investigational SARS-CoV-2 vaccine [11]. It has been observed that humans with positive SARS-CoV-2 antibody test are initially more likely to have positive nucleic acid amplification test (NAAT) test, consistent with prolonged RNA shedding. With time, they were less likely to test positive with the NAAT, indicating there is immunity developed over time [12].

Zhou *et al*. described a case with lower antibodies to SARS-COV-2 during the reinfection [13]. This suggests that a patient that developed an initial mild infection may be less likely to be protected from a reinfection if the antibody response was not adequate. Another cause may be environmental exposure to surfaces and air contaminated with the virus. Lei *et al*. found evidence of environmental contamination with the virus in isolations wards where patients who had recovered from an acute COVID-19 infection were being kept [14]. This is relevant in our patient who resided in a nursing home and where frequently residents recovering from COVID-19 are kept together in isolation in shared rooms. COVID-19 surveys in nursing homes around the world have shown high infection and high mortality rates and have shown to be more prevalent in larger nursing homes [15, 16].

An important potential cause for reinfection, and one that is worse in severity than the initial infection, is reinfection caused by a genetically distinct virus. A plethora of research is being conducted globally on this novel coronavirus to try to obtain knowledge on the virus, including how reinfection might occur. The initial thought in the first few months of the COVID-19 pandemic was that a second infection, or reinfection, was merely just a continuation of the first. However, as reinfection cases continued to increase, the idea of a variant strain of the virus causing the reinfection became more likely. Studies have now suggested that there is variation in the genomes of separate infections within the same individual. In one case study, genomic analysis was done in a patient with two positive PCR tests for SARS-CoV-2 roughly 2 months apart, and separated with two negative PCR tests between them. Genetic discordance of the two SARS-CoV-2 specimens was greater than could be accounted for by short-term *in vivo* evolution and, therefore, suggested that the patient was likely infected by SARS-CoV-2 on two different occasions with a genetically distinct virus [17]. These distinct SARS-2-CoV-2 virus lineages might also have a higher rate of transmissibility, which can lead to reinfection more easily [18].

As a case report, it is difficult to demonstrate the postinfectious course of patients with COVID-19, and study the duration of immunity in this cohort of patients. Cohort studies will be better equipped to answer these questions.

## Conclusion

The SARS-CoV-2 virus causing the novel COVID-19 infection has continued to raise multiple questions as it has been studied. This case report is highly relevant given the increasing number of infections we are continuing to observe. Based on the observations made in this case, it is not clear that an initial infection and recovery provides prolonged immunity beyond 2 months, and even if antibodies are present, it does not guarantee an attenuated course during reinfection. Therefore, vaccination plays an important role in prevention. Long-term cohort studies will be needed to study the factors behind reinfection.

## Data Availability

Not applicable.
